# Education Research: A National Pediatric Neurology In-Training Examination and its Relationship to Royal College of Physicians of Canada Certification Success

**DOI:** 10.1212/NE9.0000000000200347

**Published:** 2026-08-20

**Authors:** David J.A. Callen, Michael J. Del Bel, Eralda Gjika, Farhan Bhanji, Susan Humphrey-Murto, Sunita Venkateswaran

**Affiliations:** 1 Department of Pediatrics, McMaster University, Hamilton, Canada;; 2 Exam Quality and Analytics, Royal College of Physicians and Surgeons of Canada, Ottawa;; 3 Department of Pediatric Critical Care Medicine, McGill University, Montreal, Canada;; 4 Department of Medicine, University of Ottawa, Canada; and; 5 Department of Pediatrics, Western University, London, Canada.

## Abstract

**Background and Objectives:**

In Canadian pediatric neurology, there has historically been no standardized national progress examination aligned with the Royal College of Physicians and Surgeons of Canada (RCPSC) certification examination. The primary objective of this study was to develop such an examination—called the National InTraining Exam for Canadian Pediatric neurology residents or National In-Training Examination for Canadian pediatric neurology residents (NITECaP)—and collect evidence for its utility by examining its association with performance on the RCPSC pediatric neurology written examination. Secondary objectives were to determine whether correlations varied by postgraduate year (PGY) level and whether NITECaP scores predicted RCPSC examination success.

**Methods:**

This retrospective cohort study analyzed examination data collected between 2014 and 2024 from all 8 Canadian pediatric neurology training programs. NITECaP is a nationally administered, 3-hour, short-answer question examination modeled after the RCPSC pediatric neurology written examination and mapped to RCPSC objectives of assessment. Residents from PGY1 to PGY5 were eligible to participate. Data were included if participants completed at least one NITECaP during residency and subsequently attempted the RCPSC pediatric neurology examination. Pearson correlations were calculated between NITECaP scores at each PGY level and RCPSC examination scores. Logistic regression models were used to evaluate whether NITECaP performance predicted RCPSC examination outcomes (pass/fail).

**Results:**

A total of 179 residents completed the NITECaP, and 135 of these subsequently completed the RCPSC pediatric neurology examination. Positive correlations were observed between NITECaP and RCPSC examination scores for PGY2 through PGY5 residents, with the strongest association in PGY5 (*r* = 0.618, 95% CI 0.490–0.720). Correlations were not statistically significant for PGY1 residents. Logistic regression models demonstrated that NITECaP scores in PGY2, PGY4, and PGY5 significantly predicted RCPSC examination success, with the highest predictive accuracy in PGY5 (95.6%). PGY5 residents scoring ≥45% on the NITECaP had a high likelihood of passing the RCPSC examination.

**Discussion:**

Performance on the NITECaP is positively associated with RCPSC pediatric neurology examination performance and can predict examination success, particularly in senior trainees. These findings support the use of NITECaP as a valid national progress test to inform resident feedback, identify trainees at risk, and support program-level benchmarking in pediatric neurology training.

## Introduction

Over the past decade, Canadian postgraduate medical education has transitioned from a traditional time-based apprenticeship model to Competency-Based Medical Education (CBME).^1,2^ CBME emphasizes the achievement of clearly defined competencies and observable abilities rather than training duration alone, with progression based on demonstrated performance, frequent assessment, individualized learning, and staged attainment of clinical milestones required for independent practice.

In CBME, multiple low stakes assessments should be available to help learners move toward competency.^3^ This can be facilitated with progress testing, a form of assessment in which a test is administered repeatedly to learners at different stages in their training so that their progress can be monitored over time.^4,5^

With the implementation of CBME in the Canadian Neurology Programs in 2020, progress testing has taken on an even more important role. Before 2012, Canadian pediatric neurology trainees participated in the American Academy of Neurology (AAN) Residency In-service Training Examination (RITE) in which only a small percentage of the examination was pediatric neurology focused and the format was multiple choice. This was not reflective of the Canadian Pediatric Neurology certification examination.

The Canadian Pediatric Neurology Royal College examination has 3 different parts—2 written short-answer question examination (3 hours each) and one Observed Structured Clinical Examination (OSCE). One written component comprises only pediatric neurology content and is challenged only by pediatric neurology residents. The other written component comprises both adult and pediatric neurology content, and it is challenged by both adult and pediatric neurology residents. The OSCE comprises a mixture of stations that are pediatric neurology focused and stations that are common to both adult and pediatric neurology.

The success rate at the RCPSC Certification examination in pediatric neurology is approximately 90% for Canadian trained residents.However, every year, a few trainees are unsuccessful. There are few standardized and objective metrics to guide which trainees may require more assistance and in which areas this support is required.

As of today, there are 8 direct entry Canadian pediatric neurology training programs and select centers with a 3-year fellowship program after General Pediatric training. These programs range in size from 1 to 12 resident(s)/fellow(s). Each program has a unique educational curriculum modified to suit their local circumstances, but all based on the objectives of training set out by the Royal College of Physicians and Surgeons of Canada (RCPSC). Thus, each resident has a unique experience dependent on where they choose to train, but all are expected, by the end of training, to have covered all of the important objectives and, as such, emerge with similar competence and skill from any program across the country.

Small programs face the challenge of developing high-quality progress tests. In an effort to address this problem, the National In-Training Examination for Canadian pediatric neurology residents (NITECaP) was instituted in the 2013–2014 academic year by a combined effort from 3 Canadian pediatric neurology residency program educators (University of Ottawa, McMaster University, University of Alberta) and the president of the Canadian Association of Child Neurology.

Our objectives when designing this in-training examination were to (1) allow residents to compare their acquisition of knowledge to peers across Canada; (2) allow program directors to use objective information to identify trainees at risk of failing the final RCPSC written examination and provide them with support to allow for a greater probability of success; and (3) provide program directors with comparisons between the performance of residents in their training program to the performance of residents in other programs across Canada to identify and rectify areas of program weakness.

The purpose of this study was to collect validity evidence for a national pediatric neurology progress examination. Our first hypothesis was that there would be positive correlations between NITECaP scores and the RCPSC scores, with larger correlations for residents in the final years of residency in comparison with residents in early years of their training. We also hypothesized that the NITECaP scores would predict the likelihood of success on the RCPSC examination.

## Methods

The NITECaP oversight committee consisted of 2 primary investigators (S.V. and D.J.A.C.). All 8 pediatric neurology programs in Canada were approached and agreed to participate in the national examination project. Modern validity theory was used as a guiding framework in the development of NITECaP to ensure information related to content, response process, internal structure, correlational, and consequential sources of evidence were collected.^6^Statistical analyses were based on previous work to evaluate a progress examination for internal medicine residents in correlation to their performance on the RCPSC examination.^7^

### Standard Protocol Approvals, Registrations, and Patient Consents

Approval was obtained through the University of Ottawa as a continuous quality improvement study. Patient consent was not required.

### Examination Content, Format, and Delivery

The NITECaP examination's development, content, and delivery were modeled after the current RCPSC pediatric neurology short answer question examination. Specifically, the NITECaP blueprint was developed and based on RCPSC pediatric neurology objectives of assessment.^8^ The examination consisted of 250 marks worth of short answer questions. This usually amounted to between 45 and 55 questions, each worth a variable number of marks each. Residents were given 3 hours to complete the examination.

Each year, all Canadian pediatric neurology training programs were asked to generate new questions—questions from previous years were never reused. Each program was responsible for 25 marks of questions worth of questions from 1 or 2 specific topic category (e.g., movement disorders, neuromuscular disorders, epilepsy, etc). The proportion of the total marks assigned to each topic category was determined according to the Royal College blueprint (e.g., movement disorders—15 marks; neuromuscular disorders—25 marks). The number of questions that were created to sum to this subtotal was left up to the question creator (e.g., movement disorders—3 questions worth 5 marks each; neuromuscular disorders—3 questions worth 5 marks each, one question worth 7 marks, one question worth 3 marks). Within each topic category, the questions generated variably covered epidemiology, pathophysiology, anatomy, diagnosis, test interpretation, test ordering, management, and prognosis. No specific instructions were given for the proportion of each of these.

Questions were intentionally authored by general pediatric neurology faculty rather than subspecialists to ensure alignment with the expected knowledge base of a practicing pediatric neurologist. All question authors were provided with guidelines on creating questions in the RCPSC format.^9^ The questions and their model answers were reviewed by SV and DC and, when necessary, altered to maintain a uniform RCPSC format. The draft examination was attempted by 2 recent graduates to ensure question suitability (i.e., phrasing, content, level of difficulty, etc). Their feedback was then incorporated into the examination.

Starting in 2021, difficulty ratings were determined for each submitted question (the training level at which the majority of residents at this level of training would be expected to get the question correct) to help maintain a distribution of questions at all difficulty levels. The mean target level was year 4 of residency training. If the mean difficulty was too high or too low for the entire examination, alterations were made in the difficulty of the questions to achieve an appropriate mean.

The examination was offered as an additional training option to all Canadian pediatric neurology residents from postgraduate year (PGY) 1 to 5 of training. In most programs, it was mandatory for all residents in years 3 to 5 of training, while participation was optional in years 1 and 2. Other programs strongly encouraged residents to participate from the very beginning of their residency. One week was allocated each year for the examination to be written by all sites. The examinations were written in the presence of a proctor over a 3-hour time limit. Consent was obtained from all residents at each sitting acknowledging that scores may be used in an anonymized manner for educational research and quality assurance. Between 2014 and 2020, the blinded examination was sent to each site, printed, written in a physical form, and then sent back to the central site using only blinding numbers as identification. At the central site, all examinations were scanned into a digitized form and sent to the question creators for marking. Starting 2021, the examinations were generated as a Google Form and completed by the resident online, still using a blinding number to identify the resident.

Each question was scored by the same individual who created the question (e.g., questions 4, 7, 24, 48 were created by program X were also scored by the question creator at program X). All scorers were provided with a tip sheet on how to score in an unbiased manner consistent with the Royal College scoring methods, but otherwise, no formal training was provided. Scores were collated at the central site in a blinded manner. Once all questions had been scored and those scores received at the central site, the data were unblinded and individual results and program results were generated and disseminated. No questions were omitted from the examination based on cohort performance. No normalization was performed on the data.

Each resident received their score for each question, each subtopic, and their overall examination score. All scores were displayed compared with that of their national peers at each level of training. Each program received their resident scores with anonymized comparisons with other programs and residents at the same level of training. Programs also received data about how their residents performed in each topic category compared with peers across the country. Program directors were advised to review the examination with their residents individually or as a group at their discretion.

### Data Analysis

Analyses included an existing examination data set from a 10-year period (2014–2024). To protect participant confidentiality, the data set was cross-referenced with RCPSC records and subsequently deidentified, with PGY level being the only retained identifier. Data were only included in statistical analyses if participants completed the RCPSC pediatric neurology examination and at least one iteration of the NITECaP during their residency training. All statistical analyses used a statistical significance level of α = 0.05.

The relationships between NITECaP and RCPSC examination scores at each PGY level of training were explored using Pearson correlations and interpreted using Cohen guidelines: small (*r* = 0.10), medium (*r* = 0.30), and large (*r* = 0.50).^10^

For the group analyses, total examination scores were compared using a univariate ANOVA with PGY level (i.e., 1–5) and administration year (i.e., 2014 to 2024) as factors. This analysis was used to determine whether scores were higher for residents of increasing seniority. Logistic regression models were generated to identify whether performance on the NITECaP in different PGY levels could predict the likelihood of success (binary outcome of pass/fail) of residents on the RCPSC pediatric neurology examination. Predictive probability curves were derived for each PGY cohort model.

### Data Availability

Deidentified NITECaP scores, sample examination materials, and the statistical analysis plan can be shared for the purposes of education until 2036 by emailing the corresponding author. Requests will be reviewed to ensure consistency with the original educational and quality improvement objectives of the study. Data will be shared in deidentified form only. RCPSC data cannot be shared because of confidentiality.

## Results

**Figure 1:**
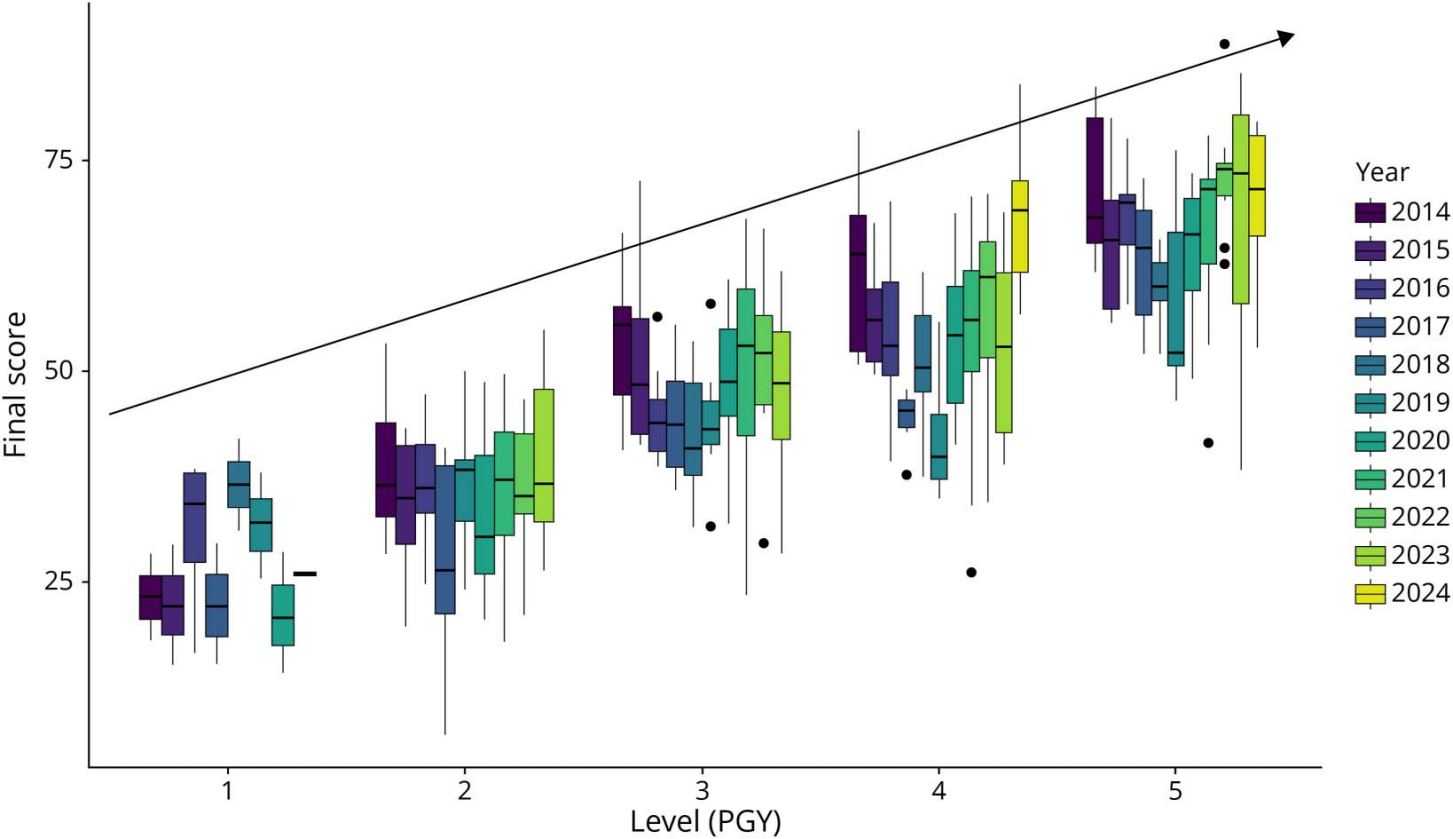
NITECaP Scores (Percentages) Over 10-Year Period for Each of the 5 PGY Levels PGY = postgraduate year

Between 2014 and 2024, 179 residents wrote the NITECaP and 135 completed the RCPSC pediatric neurology examination at the time of our analyses (Figure 1; Table 1). Of the 135 residents who completed the RCPSC, the following percentages completed the NITECaP during each PGY level: 15.6% in PGY1, 55.6% in PGY2, 71.1% in PGY3, 81.5% in PGY4, and 84.4% in PGY5.

**Table 1:** Sample Sizes, Means Exam Scores, and Standard Deviations for the NITECaP at all 5 Levels of Postgraduate Year of Training and the RCPSC Pediatric Neurology Written Examination

Data set	Sample size (n)	Mean exam score (SD)
PGY1	21	26.65 (8.17)
PGY2	75	34.90 (9.72)
PGY3	96	48.09 (9.51)
PGY4	110	54.76 (11.26)
PGY5	114	66.42 (9.95)
RCPSC	135	77.33 (6.78)

Abbreviations: PGY = postgraduate year; RCPSC = Royal College of Physicians and Surgeons of Canada.

### Correlational Analyses

All PGY cohorts had statistically significant positive correlations between their NITECaP scores and their RCPSC pediatric neurology examination scores, except PGY1 (Table 2). The largest correlation was between PGY5 and the RCPSC examination (*r* = 0.618, *p* < 0.001).

**Table 2:** Correlations Between the NITECaP Scores for Each PGY Level and the RCPSC Pediatric Neurology Written Examination

PGY cohort	Sample size	Correlation with RCPSC	T-statistic^a^	Confidence interval	*p* Value
PGY1	21	0.366	1.72 (19)	−0.077 to 0.689	0.102
PGY2	75	0.546	5.57 (73)	0.364 to 0.688	**<0.001**
PGY3	96	0.484	5.36 (94)	0.314 to 0.624	**<0.001**
PGY4	110	0.495	5.92 (108)	0.339 to 0.624	**<0.001**
PGY5	114	0.618	8.33 (112)	0.490 to 0.720	**<0.001**

Abbreviation: PGY = postgraduate year.

Statistically significant *p* values are denoted in bold.

a Degrees of freedom are listed in parentheses.

### Regression Analyses

Data from PGY1 were not used to generate a predictive model, given the absence of a significant correlation with the RCPSC examination scores and small sample size (Table 2). Logistic regression models demonstrated statistical significance for predicting success on the RCPSC pediatric neurology examination based on scores in PGY2, PGY4, and PGY5 (Table 3).

**Table 3:** Logistic Regression Models for Predicting Outcome on the RCPSC Pediatric Neurology Written Examination Using the Scores of the NITECaP for Each PGY Level

PGY cohort	Cohort description	Standard error	Odds ratio with 95% confidence interval	*p* Value	Model accuracy (%)
PGY2	Pass: 69, fail: 6 n = 75	0.06	1.15 (1.05–1.31)	**0.011**	90.7
PGY3	Pass: 89, fail: 7 n = 96	0.04	1.07 (0.99–1.18)	0.111	92.7
PGY4	Pass: 103, fail: 7 n = 110	0.06	1.20 (1.00–1.38)	**0.002**	92.7
PGY5	Pass: 107, fail: 7 n = 114	0.06	1.22 (1.10–1.40)	**0.001**	95.6

Abbreviation: PGY = postgraduate year.

Statistically significant *p* values are denoted in bold.

**Figure 2:**
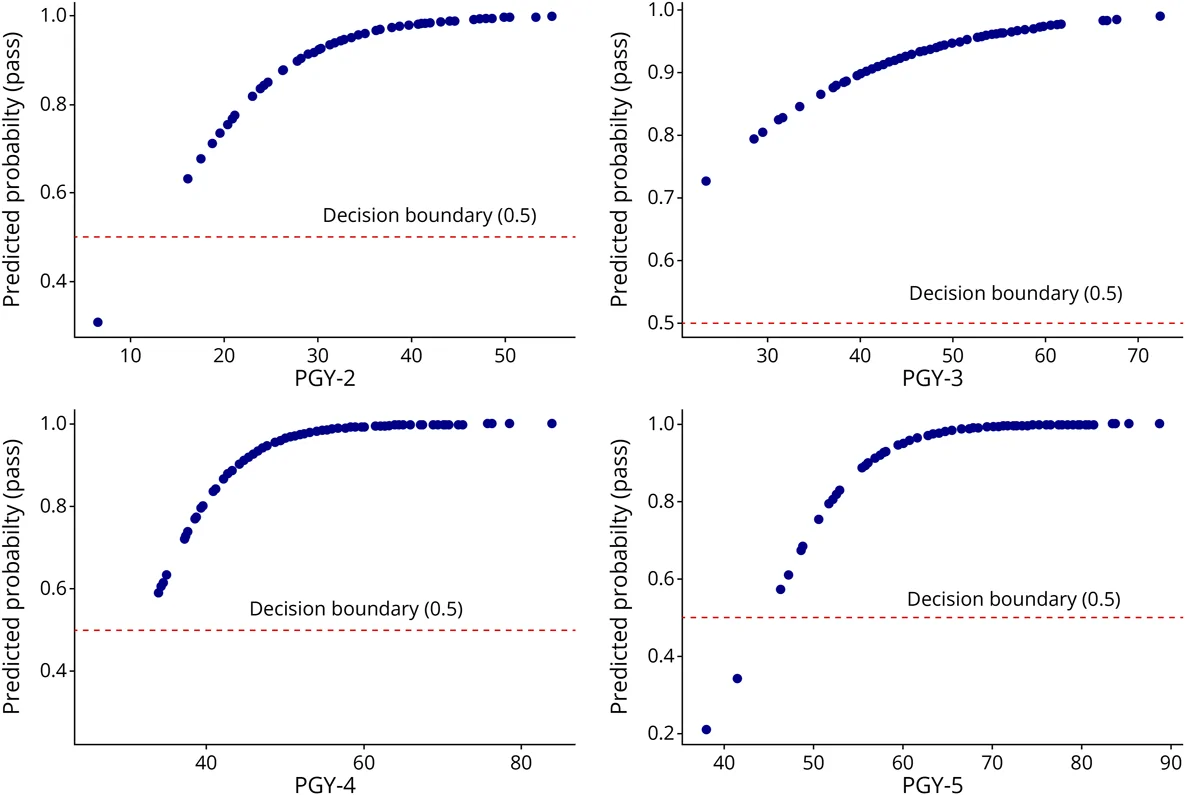
Logistic Probability Curves for Determining Success on the RCPSC Pediatric Neurology Written Examination (<0.5 Fail, >0.5 Pass) Using the Scores (Percentages) on the NITECaP for Each PGY PGY = postgraduate year; RCPSC = Royal College of Physicians and Surgeons of Canada.

Although all models had >90% accuracy, as expected, the regression model using PGY5 data had the best accuracy (95.6%, *p =* 0.001). Specifically, residents in PGY5 who completed the NITECaP and scored a minimum of 45.0% had a very high likelihood of success on the RCPSC examination (Figure 2).

## Discussion

Our results demonstrated that performance on the NITECaP can be used to provide residents with objective insights into predicting performance on the RCPSC pediatric neurology examination, thus supporting our hypotheses. Specifically, there were positive correlations between the NITECaP and RCPSC pediatric neurology examination scores, with larger correlations for residents in the final years of residency in comparison with residents in early years of residency. Furthermore, the NITECaP scores were able to predict the likelihood of success on the RCPSC examination. Our logistic regression model indicated that, for PGY5 residents who completed the NITECaP and scored a minimum of 45.0%, had a very high likelihood of passing the RCPSC pediatric neurology examination. This information can be used by program directors and competence committees to determine examination readiness and the need for additional support through enhanced educational plans during residency training.

We hypothesize that the lack of correlation between scores in a resident's third year of training and success at the Royal College examination could be due to a number of factors. It has been our experience that most residents begin to participate in formal study groups starting in their fourth year of training. Thus, in third year, there are likely candidates who score poorly who, with additional focus on semantic knowledge acquisition through study groups and textbook reading, would significantly improve their performance. Individual learning curves may also offer an explanation—we might hypothesize that the PGY3 year residents are beyond the latent phase (where performance increase is minimal as foundational knowledge is acquired), where the rate of learning is accelerating. But since each learner may be at a different point on the curve, the variability in performances between individuals may be contributing to the lack of correlation.^11^ Also, in many programs, the first few years of training are focused on foundational knowledge in pediatrics and adult neurology, whereas the final years of training are more focused on pediatric neurology and its subspecialities. It may be that, in the third year, residents have not received the detailed training in pediatric neurology yet, and as such, there is much more variability in test performance that resolves with the focused exposure that occurs in their final years.

In smaller residency programs such as pediatric neurology, residents have minimal opportunities to interact with other residents at the same training level. This creates challenges for both the resident and the faculty to determine whether they have met adequate benchmarks as they progress in training. Progress testing offers both an opportunity for single point and longitudinal benchmarking throughout the training program.^12^

Progress testing was initially developed in the 1970s to better assess student-directed, problem-based learning methods that were being introduced at that time.^5^ This type of testing is meant to provide longitudinal, curriculum-independent assessments. Rather than encouraging memorization, progress tests encourage the tenet of life-long learning aiming for mastery of a subject. This assessment for learning rather than assessment of learning^7,13^ concept does not expect students to study for the examination and does not expect junior learners to answer all the questions. Cross-institutional progress testing has been valuable in the medical school curriculum both nationally and internationally allowing resources to be pooled to conduct high-quality, valid examinations.^14-16^

With postgraduate medical education in Canada recently transitioning to CBME, more in-training assessments are necessary to continually evaluate a resident's progress. The hopes are that this fosters an environment of self-directed life-long learning, allows learners to identify difficulties early on in training and allows educators to individualize learning needs. The goals of NITECaP align extremely well with the Royal College CBME objectives, years before the initiation of CBME in Canadian pediatric neurology training programs.

Our progress test aimed at assessment of the medical expert in the subject of pediatric neurology and the improvement of knowledge with ongoing training. This test is meant to complement the assessments of the other CanMEDs roles which may be evaluated with daily clinical encounter evaluations, entrustable professional activity observations, OSCEs, 360° assessments, etc. and not as a stand-alone evaluation. Advantages of this examination format include pooled resources, avoidance of item origin bias, objective assessments, and individualized feedback for both the trainee and the institution.

An objective of this progress test was to see whether it was generalizable to the outcome on the high-stakes RCPSC pediatric neurology examination. Previous studies have demonstrated a significant correlation between performance on the AAN RITE for both adult and pediatric neurology trainees and performance on the American Board of Psychiatry and Neurology examination.^17^ However, clinical pediatrics only made up 10% of the RITE and the examination format is not similar to the RCPSC pediatric neurology examination. In addition, only senior neurology residents were evaluated in the aforementioned study, whereas we evaluated residents at all levels to look at the preexisting “risk” for failure at the RCPSC. Although not in neurology, a similar structured study was conducted to assess the predictability of performance on the RCPSC internal medicine examination using OSCE progress test scores completed during resident training.^7^ Compared with this study, they found similar and supportive results, with positive correlations between the progress test and RCPSC internal medicine examination scores. Furthermore, their logistic regression models were predictive of performance on the RCPSC examination.

The NITECaP was found to be both valid and reliable for its purpose in providing an assessment with feedback for pediatric neurology residents in training. We have demonstrated a clear progression in child neurology–specific knowledge over the 5 years of postgraduate resident training.^16^

Multiple surveys of residents and program directors have been performed over the past 12 years of the NITECaP. From a resident perspective, there has been consistent positive feedback about insight into progression in training, comparison with peers across the country, and exposure to the Royal College experience. Most residents and program directors map the score of each resident from year to year to determine the slope of their acquisition of pediatric neurology knowledge and compare this with previous resident cohorts to ensure that their trajectory predicts success at the Royal College or whether it may indicate a need for additional support and focused training. Before the NITECaP, there were a number of resident comments about feeling like they had no idea whether they were learning the things they were supposed to know and how that compared with peers at the same stage of training across the country. With the advent of the NITECaP, residents were able to determine not only their performance to their local peers but also to peers across Canada. Finally, before the NITECaP, many training programs had residents write the American Academy of Neurology Resident In Training Exam, which was useful, but was not reflective of the Royal College experience in pediatric neurology (because the examination format and content was very different). With the NITECaP, residents were able to experience a version as close as possible to the “real thing” each year so that, when challenging the actual Royal College examination, there were no surprises.

From a program perspective, the information from the NITECaP could be used for continuous quality improvement. Metrics were provided each year about how their residents performed compared with residents across the country with respect to scores at each level of training, slope of accrual of knowledge in their program compared with other programs, and a detailed breakdown of which topics were done better than average and which were problematic. This information could be used to review the program curriculum and make changes to increase resident training in areas where the program was consistently performing lower than the national average.

This study had several limitations that should be considered when interpreting the findings. First, given that pediatric neurology is a smaller residency program, sample sizes used in analyses were small and inconsistent across levels, with gradual increases in test cohort sizes as residents progressed through the training. Moreover, candidates were not always repeat test takers; only 13 of 135 participants completed the practice examination at all 5 levels in addition to the RCPSC pediatric neurology examination, limiting the ability to conduct longitudinal or within-subject analyses. Second, the number of candidates who failed the RCPSC pediatric neurology examination was limited over the 10-year period (n = 15); in contrast, pass rates for the NITECaP were substantially lower across all PGY levels, which may reflect differences in examination difficulty or participant preparedness. Finally, the NITECaP is not mandatory for residents in their training, which may have led to an underestimation of correlations because of self-selection bias and variability in candidate motivation.

The NITECaP has been successfully implemented in all 8 pediatric neurology training programs in Canada since 2014 and was formally endorsed by the Canadian Association of Child Neurology in 2019. The results of this study demonstrate the validity and value of the NITECaP for residents in all years of training. The NITECaP has, and will continue to provide residents, program directors, and competence committees with objective insights into determining examination readiness and, if needed, tailoring of training to better support residents during their development. The results from this study will be of interest to other small programs both nationally and internationally and serve as a model for those who are transitioning to or have already transitioned to CBME.

Our success stems from nationwide engagement from stakeholders—the programs, the residents, and the national association. An educational subcommittee has been created within the CACN based on the interested in participating in the NITECaP administration. This committee hopes to continue fostering nationwide engagement in resident education and include specific workshops in upcoming national meetings on question writing, modifying the current examination bank into methods of evaluation specific to the pediatric neurology CBME curriculum.

## Glossary

AAN: American Academy of Neurology
CBME: competency based medical education
NITECaP: National In-Training Examination for Canadian pediatric neurology residents
OSCE: Observed Structured Clinical Exam
PGY: postgraduate year
RCPSC: Royal College of Physicians and Surgeons of Canada
RITE: Residency In-service Training Examination
